# PhageDx™ *E. coli* O157:H7 Assay in Ground Beef Level 3 Modification to Add 375 g Beef Trim and New Method Procedure for 25 g Ground Beef: AOAC *Performance Tested Method*^SM^ 081601

**DOI:** 10.1093/jaoacint/qsab039

**Published:** 2021-03-18

**Authors:** Stephen Erickson, Jose Gil, Minh Nguyen

**Affiliations:** 1 Laboratory Corporation of America®/MedTox, 402 County Road D West, St. Paul, MN 55112, USA; 2 Laboratory of America Corporation®/National Genetics Institute, 2440 Sepulveda Blvd., Suite 235, Los Angeles, CA 90064, USA

## Abstract

**Background:**

ThePhageDx^™^ *E. coli* O157:H7 Assay originally required a 5 h enrichment period and a 10 mL test sample for 25 g ground beef. The proposed method modifications seek to include beef trim (375 g) matrix and a new method procedure for 25 g ground beef comprised of 6–7 h enrichment and 1 mL test sample.

**Objective:**

To validate the method modifications for PhageDx *E. coli* O157:H7 Assay to include beef trim (375 g) matrix and modify enrichment time and test sample size for 25 g ground beef.

**Method:**

For both matrixes, pre-warmed tryptic soya broth (TSB; 42 ± 1°C) was added in a 3:1 (media:sample size) ratio, blended, and enriched for 9–10 h (beef trim, 375 g) or 6 h (ground beef, 25 g) at 42 ± 1°C. One milliliter samples were transferred to a microfuge tube, centrifuged, and the supernatant removed. The pellet was resuspended in 0.2 mL of TSB and phage reagent was added. Samples were incubated for 2 h at 37 ± 1°C. After infection, the sample was centrifuged, and 150 µl of the supernatant was transferred to a 96-well plate. Then, lysis buffer and luciferase substrate were added and the sample read on a luminometer to determine the presence or absence of *E. coli* O157:H7.

**Results:**

No significant differences were found between the PhageDx *E. coli* O157:H7 Assay method modifications and the U.S. Department of Agriculture Food Safety and Inspection Service (FSIS) *Microbiology Laboratory Guidebook* (MLG) reference method.

**Conclusions:**

The independent study demonstrated that the PhageDx *E. coli* O157:H7 Assay method modifications meet the qualifications for Performance Tested Method ^SM^ status.

**Highlights:**

The PhageDx *E. coli* O157:H7 Assay is a rapid detection method capable of detecting *E. coli* O157:H7 in 25 g ground beef and 375 g beef trim samples in as little as 8.5 h and 11.5 h total turn around time, respectively.

## General Information

*Escherichia coli* O157:H7 is a pathogenic serotype of *E. coli* that is classified as an enterohemorrhagic *E. coli* (EHEC) and is one of the most common pathogenic bacteria in food microbiology. One of the most common sources of *E. coli* O157:H7 within the food industry is contaminated beef products, from raw ground beef to undercooked steaks. *E. coli* O157:H7 is related with illnesses that range from mild gastroenteritis to life-threatening hemorrhagic colitis and hemolytic uremic syndrome. *E. coli* O157:H7 carries a number of pathogenic factors, such as Shiga toxins. Pathogenic E. coli O157:H7 also has a low infective dose estimated to be between 1–50 cells. All handling of any pathogen or contaminated meat must be done in a microbiology facility with appropriate containment devices and staffed with trained technicians in accordance with “Biosafety in Microbiological and Biomedical Laboratories” from the U.S. Department of Health and Human Services (2).

## Principle of the Method

The PhageDx™ E. coli O157:H7 Assay is based on the infection of *E. coli* O157:H7 by a bacteriophage and replication of the infecting bacteriophage within its specific host. Bacteriophages demonstrate a high specificity for their bacterial host and are capable of replicating within their host quickly to high numbers. The recombinant phage used in the PhageDx E. coli O157:H7 Assay also expresses a luciferase reporter during replication. The presence of *E. coli* O157:H7 is determined by incubating the lysate with the appropriate luciferase substrate and detecting emitted light in a luminometer. An absence of detected light indicates that no *E. coli* O157:H7 are present in that sample. An advantage of this system is that only viable bacteria cells are detected as bacteriophage only replicate in living cells.

## Scope of Method

- *Target organism.*—*E. coli* O157:H7.
- *Matrix.*—Fresh raw ground beef, 70% lean (25 g); raw beef trim, containing >30% fat content (375 g).
- *Summary of validated performance claims*.—Performance equivalent to that of the U.S. Department of Agriculture (USDA) Food Safety and Inspection Service (FSIS) *Microbiology Laboratory Guidebook* (*MLG*) Chapter 5.09 “Detection, Isolation and Identification of *Escherichia coli* O157:H7 from Meat Products and Carcass and Environmental Sponges” (1).

## Definitions

- *Probability of detection (POD).*—The proportion of positive analytical outcomes for a qualitative method for a given matrix at a given analyte level or concentration. POD is concentration dependent. Several POD measures can be calculated: POD_R_ (reference method POD), POD_C_ (confirmed candidate method POD), POD_CP_ (candidate method presumptive result POD), and POD_CC_ (candidate method confirmation result POD).
- *Difference of probabilities of detection (dPOD).*—Difference of probabilities of detection is the difference between any two POD values. If the confidence interval (CI) of a dPOD does not contain zero, then the difference is statistically significant at the 5% level.

## Materials and Methods

### Test Kit Information

- *Kit name*.—PhageDx *E. coli* O157:H7 Assay.
- *Catalog Nos*.—5003 (25 g), 5005 (375 g).
- *Ordering information*.—Not applicable. For internal use at Laboratory Corp. of America only.

### Test Kit Components

- *PhageDx O157:H7 recombinant phage.*—Part. No. 3009, 12 tubes containing 150 µL phage solution.
- *Lysis buffer.*—Part. No. 3002, 12 tubes containing 100 µL lysis buffer.
- *Assay buffer.*—Part. No. 3003, 12 tubes containing 500 µL assay buffer.
- *Luciferase substrate.*—Part. No. 3004, 12 tubes containing 10 µl luciferase substrate.
- *96-well break-apart plate.*—Part. No. 3005, one pouch containing break-apart plate (8 wells × 12 strips).
- *One package insert.*—Part. No. 3007 (25 g) or 3008 (375 g).

### Additional Supplies and Reagents

- *Filter blender bags for 25 g and 375 g samples.*
- *Microfuge tubes (1.5 mL).*
- *Blender bag and tube racks.*
- *Tryptone soya broth (TSB).*—Thermo Fisher Scientific, cat. No. OXCM0129B.
- *Sample pipettor (2–5 mL).*
- *Sterile, filtered pipet (2–5 mL)*.
- *Adjustable single channel pipettor (10 µL–1 mL) and appropriate sterile filter tips.*
- *Appropriate personal protective equipment.—See* https://www.cdc.gov/labs/pdf/CDC-BiosafetyMicrobiologicalBiomedicalLaboratories-2009-P.PDF (2).
- *Dynabeads^®^ anti-O157 magnetic particles.*—Life Technologies (Thermo Fisher Scientific), cat. No. 71004.
- *CHROMagar^™^ O157 plates.*—BD (Franklin Lakes, NJ), cat. No. 214984.
- *Remel^™^ Wellcolex^™^ E. coli O157:H7 Rapid latex agglutination test.*—Thermo Fisher Scientific, cat. No. R30959601.
- *Phosphate buffered saline (PBS).*

### Apparatus

- *Homogenizer.*—Seward Stomacher^®^ 400 and 3500 or equivalent.
- *Air incubators.—*Capable of 37 ± 1°C and 42 ± 1°C.
- *Microfuge.—*Capable of 10 000 × *g*.
- *Vortex.*
- *Promega GloMax^®^96 or Navigator luminometer.*
- *Personal tablet/computer for luminometer control and data analysis.*

### Safety Precautions

- The PhageDx *E. coli* O157:H7 Assay involves the enrichment of samples which may contain human pathogenic *E. coli* and have the potential for contamination with subsequent handling of those samples. This method should be conducted by properly trained laboratory personnel in a suitable microbiology laboratory in accordance with “Biosafety in Microbiological and Biomedical Laboratories”, U.S. Department of Health and Human Services (2). Care should be taken when handling the sample and reagents while performing the method.
- Materials and reagents provided in the PhageDx *E. coli* O157:H7 Assay are not considered hazardous if used according to the assay method. Please review the Material Safety Data Sheet prior to performing the assay.
- Follow all relevant guidelines and laboratory protocols while performing the assay and manufacturer’s equipment instructions.

### General Preparation

- Use aseptic techniques.
- Prepare TSB media according to manufacturer’s instructions.
- Before using the reagents, flick or spin the tube to collect all of the solution at the bottom of the tube.
- Due to the short enrichment times, it is vital to maintain the temperature of the sample and TSB media used in the incubation.
- Bring the raw beef sample to room temperature before continuing with the assay.
- Before adding the pre-warmed TSB to the sample, confirm that the media and incubator are warmed to 42 ± 1°C.
- Do not allow the pre-warmed media to cool before adding to the sample.
- Maintain the media at 42 ± 1°C in an incubator or water bath if preparing multiple samples.

### Sample Preparation

- Weigh 25 g of the raw ground beef or 375 g of beef trim and place into a filter blender bag.
- Add 75 mL (25 g raw ground beef) or 1125 mL (375 g raw beef trim) of pre-warmed (42 ± 1°C) TSB to the sample.
- Homogenize sample in a Stomacher 400 at the lowest setting for 30 s for 25 g raw ground beef or 2 min in a Stomacher 3500 for 375 g raw beef trim (or equivalent homogenizer and setting).
- Loosely close the blender bag and place in a static air incubator at 42 ± 1°C for 6–7 h (25 g ground beef) or 9–10 h (375 g beef trim) using a sample rack to keep the bags separate.
- After enrichment, remove sample bags from the incubator and mix contents of sample bag thoroughly by shaking the bag and squeezing the contents of the sample bag by hand for 30–60 sec. Immediately proceed to the next step after mixing is complete. If sample sits for 15 min or longer, mix sample again before proceeding to the next step.
- Using a pipettor with a sterile 2 mL pipet, transfer 1 mL of the sample to a sterile 1.5 mL microfuge tube. Avoid transferring fat and meat particles as much as possible.
- Centrifuge the sample at 10 000 × *g* for 1 min and then carefully decant the supernatant, leaving the pellet undisturbed.
- Completely resuspend the pellet in 0.2 mL TSB pre-warmed to 37 ± 1°C by running tube vigorously along tube rack and vortexing.
- Using a single channel pipettor and clean tip for each sample, add 15 µL of the phage solution to the resuspended sample and mix completely and gently by rotating the tube end over end. Do not vortex sample after adding phage.
- Place the sample in the 37 ± 1°C incubator for 2 h.
- After incubation, give the samples a brief vortexing and centrifuge at 10 000 × *g* for 10–20 s.
- Using a pipettor, transfer 150 µL of the supernatant of the infected sample to a single well.
- Using a clean tip for each sample, add 10 µL lysis buffer and mix thoroughly by pipetting up and down.
- Add 50 µL of the 1:50 substrate working solution to each well using a single channel pipettor. Mix thoroughly by pipetting up and down. To avoid cross-contamination, use a clean tip for each sample.
- Once all of the samples have received the substrate, place the sample plate in the luminometer, close the lid and initiate the read program.

### Interpretation and Test Result Report

- The luminometer program will display the results on the screen as relative light unit (RLU) values corresponding to the well positions of the break-away plate.
- For 25 g ground beef samples, samples positive for *E. coli* O157:H7 will have a reading value of 151 RLU or greater. Negative samples will be <150 RLU. For 375 g beef trim samples, samples positive for *E. coli* O157:H7 will have a reading value of 301 RLU or greater. Negative samples will be <300 RLU. Note samples that are positive.
- Once all of the samples have been run and analyzed, remove the plate from the luminometer and follow the manufacturer’s instructions for cleaning the instrument and shut down.

### Confirmation

- Confirm *E. coli* O157:H7 as described in USDA-FSIS MLG 5.09.
- Alternatively, confirmation of *E. coli* O157:H7 can be performed on overnight enriched cultures using immuno-magnetic separation (IMS) with particles coated with O157 antibodies (Dynabeads) and plating onto CHROMagar O157 plates. To prepare for the confirmation, allow the samples to continue enriching overnight (18–24 h total or an additional 13–19 h) at 42 ± 1°C. Remove 400 µL of the overnight culture and add to 600 µL TSB. Follow Dynabeads anti-*E. coli* O157 procedure. Briefly, add 20 µL of IMS particles to the diluted overnight culture and incubate for 10 min at room temperature. Isolate magnetic particles for 3 min with the magnet, and then wash three times with PBS, 1 mL per wash. After final wash, plate the particles onto CHROMagar O157 plates and incubate 18–24 h at 37 ± 1°C.
- Culture at least one mauve-colored colony (presumptive positive) in TSB media overnight (18–24 h) at 37 ± 1°C for serological confirmation. Presence of O157 and H7 antigens is determined using agglutination assay (Remel Wellcolex *E. coli* O157:H7). Follow the manufacturer’s instructions using 40 µL of the overnight culture. Results will confirm presence or absence of O157 and/or H7 antigens and provide confirmation for *E. coli* O157:H7.

## Validation Study

This method modification validation study was conducted under the AOAC Research Institute *Performance Tested Method* program and the *AOAC INTERNATIONAL Methods Committee Guidelines for Validation of Microbiological Methods for Food and Environmental Surfaces* (3). The matrix testing was conducted independently by Q Laboratories and included matrix studies for ground beef and beef trim.

### Matrix Study

The evaluation included matrix studies for raw ground beef and raw beef trim comparing the PhageDx *E. coli* O157:H7 Assay to the USDA-FSIS MLG 5.09 reference method. Within each sample set, there were five uninoculated samples (0 CFU/test portion), 20 low-level inoculated samples (0.2–2 CFU/test portion), and five high-level inoculated samples (2–10 CFU/test portion). The low inoculation level was designed to produce fractional positive results, those in which the candidate or reference method produced 5–15 positive results (25–75%).

The raw ground beef (70% lean) and raw beef trim (>30% fat) were purchased from a local distributor and prescreened for natural contamination of the analyte following the USDA-FSIS MLG 5.09 reference method. No natural contamination was observed. Each matrix was inoculated with a different strain of *E. coli* O157:H7; *E. coli* O157:H7 (ATCC 43895) was used for the raw ground beef and *E. coli* O157:H7 (ATCC 51657) was used for the raw beef trim. *E. coli* O157:H7 strains were grown in brain heart infusion broth and spiked into beef at fractional (0.2–2 CFU/test portion) and high (2–10 CFU/test portion) levels. To prepare the 25 g samples, a 25 g sample from each bulk test portion was directly weighed out. To prepare the 375 g test portion, a 25 g inoculated test portion was added to a 350 g test portion of uninoculated test product. After inoculation, samples were refrigerated at 4 ± 2°C for 48–72 h.

Total aerobic count was determined according to U.S. Food and Drug Administration (FDA) *Bacteriological Analytical Manual* (BAM) Chapter 3 (4). The level of *E. coli* O157:H7 in the low level inoculum and high level inoculum was determined by most probable number (MPN) on the day of analysis. For the unpaired analysis, the low level MPN was determined by evaluating 5 × 50 g test portions, the 20 × 25 g reference method test portions from the study, and 5 × 10 g test portions. The level of *E. coli* O157:H7 in the high level inoculum was determined by evaluating the 5 × 25 g reference method test portions from the study, 5 × 10 g test portions, and 5 × 4 g test portions. Each test portion was enriched with the reference method enrichment and analyzed by the reference method procedure. The number of positives from the three test levels was used to calculate the MPN using the LCF MPN calculator (5).

PhageDx *E. coli* O157:H7 Assay test portions were processed according to the product directions for use. Each 25 g ground beef portion received 75 ± 1.5 mL of pre-warmed (42 ± 1°C) TSB, was homogenized, and was enriched at 42 ± 1°C for 6–7 h. Each 375 g beef trim portion received 1125 mL of pre-warmed (42 ± 1°C) TSB, was homogenized, and was enriched at 42 ± 1°C for 9–10 h. Bacteriophage infection and signal reading were performed as described in the PhageDx *E. coli* O157:H7 Assay protocol. A sample from each enriched test portion (1 mL) was transferred to a 1.5 mL microfuge tube and centrifuged. The supernatant was decanted, and the pellet was resuspended in 0.2 mL pre-warmed (37 ± 1°C) TSB. Fifteen microliters of phage solution was added to each resuspended sample, mixed, and then incubated at 37 ± 1°C for 2 h. Following incubation, samples were briefly mixed and centrifuged, and then 150 µL from each sample was added to a clean well. After samples were lysed and substrate added, sample plates were placed in the luminometer for reading. RLU values of 151 or higher were considered positive for 25 g ground beef samples and 301 or higher were considered positive for 375 g beef trim samples. For confirmation, test portions were placed back in the incubator at 42 ± 1°C and allowed to enrich for a total of 15–24 h. Each PhageDx test portion was confirmed using the USDA-FSIS MLG 5.09 reference method. For the USDA-FSIS MLG 5.09 test portions, fresh raw ground beef and raw beef trim, test portions consisting of 25 ± 2.5 g, were enriched with 225 ± 4.5 mL of pre-warmed (42 ± 1°C) modified TSB (mTSB), homogenized by stomaching for 2 min, and incubated for 15–24 h at 42 ± 1°C. After incubation, the primary enrichment from each sample was screened using an USDA-FSIS MLG 5.09 validated lateral flow device test system (PTM 070801). Regardless of the screening result, all samples were subjected to isolation by IMS by transferring 1.0 mL aliquots of the primary enrichment to a microcentrifuge tube containing a 50 µL suspension of *E. coli* O157 immunomagnetic (paramagnetic) beads. The solution was placed onto a Labquake™ agitator and rotated for 10–15 min at 18–30°C. After rotation, the bead and sample solution were transferred to a MACS^®^ large cell separation (ferromagnetic) column and was washed four times with E buffer (pre-warmed to 18–35°C) before the final elute was collected with 1 mL of E buffer into a sterile tube. Following the IMS procedure, a 1:10 dilution and a 1:100 dilution of each IMS suspension in E buffer, were spread plated onto modified Rainbow^®^ agar (mRBA).

A 450 µL aliquot of each remaining IMS suspension was transferred into a microcentrifuge tube and mixed with 25 µL of a 1 N HCl solution. The microcentrifuge tubes were vortexed briefly and placed onto a Labquake agitator and rotated for 1 h at 18–30°C. After rotating, 475 µL of E buffer was added to each sample tube. The acid treated IMS suspension and a 1:10 dilution of this suspension in E buffer were plated onto mRBA. All mRBA plates were incubated for 20–24 h at 35 ± 2°C. After incubation, plates were observed for typical colonies. The mRBA plates containing typical colonies were tested for O157 latex agglutination and up to five isolated colonies were streaked to SBA. The SBA plates were incubated for 16–24 h at 35 ± 2°C. After incubation, SBA plates were observed for purity. Isolated colonies from SBA were confirmed positive by conducting an H7 latex agglutination test and for the presence of Shiga toxins using the USDA approved Real-Time PCR assay (PTM 091301). Final biochemical confirmations were obtained by AOAC *Official Method* **2011.17** (6).

## Results

All test results were analyzed using POD statistical analysis to 95% (CI). POD analysis is described in the AOAC INTERNATIONAL guidelines in Appendix J (3). Data from the analysis are presented in Tables 1–2.

**Table 1.** PhageDx™ *E. coli* O157:H7 Assay presumptive vs confirmed results using Reference Method MLG 5.09 confirmation procedure

Matrix	Strain	MPN/test portion^a^	N^b^	PhageDx presumptive			PhageDx (MLG 5.09 confirmed)			dPOD_CP_^f^	95% CI^g^
				x^c^	POD_CP_^d^	95% CI	x	POD_CC_^e^	95% CI		
Raw ground beef 25 g (1 mL)	*E. coli* O157:H7 (ATCC 43895)^h^	N/A^i^	5	0	0.00	0.00, 0.43	0	0.00	0.00, 0.43	0.00	−0.47, 0.47
		0.90 (0.54, 1.51)	20	13	0.65	0.43, 0.82	13	0.65	0.43, 0.82	0.00	−0.13, 0.13
		4.38 (1.71, 11.19)	5	5	1.00	0.57, 1.00	5	1.00	0.57, 1.00	0.00	−0.47, 0.47
Raw beef trim 375 g (1 mL)	*E. coli* O157:H7 (ATCC 51657)	N/A	5	0	0.00	0.00, 0.43	0	0.00	0.00, 0.43	0.00	−0.47, 0.47
		0.61 (0.33, 1.04)	20	10	0.50	0.30, 0.70	10	0.50	0.30, 0.70	0.00	−0.13, 0.13
		2.98 (1.27, 6.95)	5	5	1.00	0.57, 1.00	5	1.00	0.57, 1.00	0.00	−0.47, 0.47

a MPN is based on the POD of reference method test portions using the Least Cost Formulations MPN calculator, with 95% CI.

b N = Number of test portions.

c x = Number of positive test portions.

d POD_CP_ = Candidate method presumptive positive outcomes divided by the total number of trials.

e POD_CC_ = Candidate method confirmed positive outcomes divided by the total number of trials.

f dPOD_CP_ = Difference between the candidate method presumptive result and candidate method confirmed result POD values.

g 95% CI = If the CI of a dPOD does not contain zero, then the difference is statistically significant at the 5% level.

h ATCC = American Type Culture Collection, Manassas, VA.

^i^N/A = Not applicable.

**Table 2.** PhageDx *E. coli* O157:H7 Assay (MLG 5.09 confirmation procedure) vs MLG 5.09 method comparison results

Matrix	Strain	MPN/test portion^a^	N^b^	PhageDx result			MLG 5.09 result			${\text{dPOD}}_{C}^{f}$	95% CI^g^
				x^c^	${\text{POD}}_{C}^{d}$	95% CI	x	${\text{POD}}_{R}^{e}$	95% CI		
Raw ground beef 25 g (1 mL)	*E. coli* O157:H7 (ATCC 43895)^h^	N/A^i^	5	0	0.00	0.00, 0.43	0	0.00	0.00, 0.43	0.00	−0.47, 0.47
		0.90 (0.54, 1.51)	20	13	0.65	0.43, 0.82	11	0.55	0.34, 0.74	0.10	−0.19, 0.37
		4.38 (1.71, 11.19)	5	5	1.00	0.57, 1.00	5	1.00	0.57, 1.00	0.00	−0.47, 0.47
Raw beef trim 375 g (1 mL)	*E. coli* O157:H7 (ATCC 51657)	N/A	5	0	0.00	0.00, 0.43	0	0.00	0.00, 0.43	0.00	−0.47, 0.47
		0.61 (0.33, 1.04)	20	10	0.50	0.30, 0.70	9	0.45	0.26, 0.66	0.05	−0.24, 0.33
		2.98 (1.27, 6.95)	5	5	1.00	0.57, 1.00	5	1.00	0.57, 1.00	0.00	−0.47, 0.47

a MPN is based on the POD of reference method test portions using the Least cost Formulations MPN calculator, with 95% CI.

b N = Number of test portions.

c x = Number of positive test portions.

d POD_C_ = Candidate method presumptive positive outcomes confirmed positive.

e POD_R_ = Reference method confirmed positive outcomes divided by the total number of trials.

f dPOD_C_ = Difference between the candidate method and reference method POD values.

g 95% CI = If the CI of a dPOD does not contain zero, then the difference is statistically significant at the 5% level.

h ATCC = American Type Culture Collection, Manassas, VA.

i N/A = Not applicable.

The independent laboratory study showed that there were no differences between the PhageDx and the USDA-FSIS confirmation procedures for both 25 g ground beef and 375 g beef trim matrixes (Tables 1 and 2). All test portions that were presumptive positive by the PhageDx *E. coli* O157:H7 Assay were confirmed by USDA-FSIS MLG. There were no false negative results. The POD analysis indicated that no significant differences exist between the PhageDx *E. coli* O157:H7 Assay confirmed results and the USDA-FSIS MLG reference method results in this unpaired study (Table 2). The aerobic plate count of the beef matrix used in the study was 5400 CFU/g for the ground beef and 29 000 CFU/g for the beef trim, indicating approximately 135 000 CFU and 10 875 000 CFU, respectively, of background flora were present at the initiation of the enrichment process.

## Discussion

The original PhageDx *E. coli* O157:H7 Assay (PTM 081601) demonstrated that the assay could detect the presence of *E. coli* O157:H7 in 25 g of ground beef with a 5 h enrichment and 10 mL test sample. In this method modification study, we have included an alternative smaller test sample size (1 mL) with a longer enrichment period (6–7 h) for 25 g ground beef, and an additional matrix, beef trim (375 g) with a 1 mL test sample and 9–10 h enrichment time. A smaller sample size makes the assay more accessible to those that may not have use of a table top centrifuge. In addition, the inclusion of beef trim allows the assay to be used for testing of another matrix typically tested for presence of *E. coli* O157:H7. These modifications provide more flexibility to the user.

Independent laboratory testing demonstrated that the PhageDx *E. coli* O157:H7 Assay was able to detect *E. coli* O157:H7 at low levels in 25 g raw ground beef and 375 g beef trim, which also contained approximately 135 000 CFU and 10 875 000 CFU background flora, respectively. The results of this validation study demonstrate that the method modifications to the PhageDx *E. coli* O157:H7 Assay (PTM 081601) proposed are an effective alternative to the USDA-FSIS MLG 5.09 for the detection of *E. coli* O157:H7 in 25 g raw ground beef and 375 g beef trim samples.

## Conclusion

The proposed method modifications of the PhageDx *E. coli* O157:H7 Assay demonstrate that it is a specific, sensitive, fast, and simple method for the detection of a single viable *E. coli* O157:H7 bacterium in 25 g of raw ground beef and 375 g beef trim. By using a luciferase-expressing recombinant bacteriophage, the assay was able to detect a single bacterium after a 6–7 h enrichment (25 g ground beef) or 9–10 h enrichment (375 g beef trim) and a 2 h infection. These modifications have increased the usability of the assay by providing alternatives to the size of the test sample and expanding the matrix list to include beef trim. The PhageDx™ *E. coli* O157:H7 Assay offers shorter time-to-results compared with many other *E. coli* O157:H7 detection assays and it also detects only viable bacteria in a sample. The assay has a total of five basic steps that are essentially the same with minor changes in reagent volumes to accommodate the change in sample size: enrichment, concentration, infection, substrate addition, and signal readout, and involves approximately 30 min sample handling time. The PhageDx *E. coli* O157:H7 Assay with the proposed method modifications provides the user with a simplified, easy to use method for pathogen detection.

## Submitting Company

Laboratory Corporation of America^®^

531 South Spring Street

Burlington, NC 27215, USA

## Independent Laboratories

Benjamin Bastin, Patrick Bird, James Agin and David Goins

Q Laboratories Inc.

Cincinnati, OH 45204, USA
